# Predation-Related Costs and Benefits of Conspecific Attraction in Songbirds—An Agent-Based Approach

**DOI:** 10.1371/journal.pone.0119132

**Published:** 2015-03-19

**Authors:** Jakub Szymkowiak, Lechosław Kuczyński

**Affiliations:** Department of Avian Biology and Ecology, Institute of Environmental Biology, Faculty of Biology, Adam Mickiewicz University, Poznań, Poland; Università degli Studi di Milano-Bicocca, ITALY

## Abstract

Songbirds that follow a conspecific attraction strategy in the habitat selection process prefer to settle in habitat patches already occupied by other individuals. This largely affects the patterns of their spatio-temporal distribution and leads to clustered breeding. Although making informed settlement decisions is expected to be beneficial for individuals, such territory clusters may potentially provide additional fitness benefits (e.g., through the dilution effect) or costs (e.g., possibly facilitating nest localization if predators respond functionally to prey distribution). Thus, we hypothesized that the fitness consequences of following a conspecific attraction strategy may largely depend on the composition of the predator community. We developed an agent-based model in which we simulated the settling behavior of birds that use a conspecific attraction strategy and breed in a multi-predator landscape with predators that exhibited different foraging strategies. Moreover, we investigated whether Bayesian updating of prior settlement decisions according to the perceived predation risk may improve the fitness of birds that rely on conspecific cues. Our results provide evidence that the fitness consequences of conspecific attraction are predation-related. We found that in landscapes dominated by predators able to respond functionally to prey distribution, clustered breeding led to fitness costs. However, this cost could be reduced if birds performed Bayesian updating of prior settlement decisions and perceived nesting with too many neighbors as a threat. Our results did not support the hypothesis that in landscapes dominated by incidental predators, clustered breeding as a byproduct of conspecific attraction provides fitness benefits through the dilution effect. We suggest that this may be due to the spatial scale of songbirds’ aggregative behavior. In general, we provide evidence that when considering the fitness consequences of conspecific attraction for songbirds, one should expect a trade-off between the benefits of making informed decisions and the costs of clustering.

## Introduction

Habitat selection is a decision-making process that leads to observed patterns of spatio-temporal distribution of individuals. In this process, songbirds may use various types of social information to choose between different sites and decide where to settle, one of them being conspecific cues [1–5]. Because breeding density in a given habitat is typically correlated with that habitat’s quality [6], the presence of conspecifics may serve as a cue for individuals when making settlement decisions. For most songbirds exhibiting conspicuous territorial behavior, this information is available for both the pre- and post-breeding periods and may be acquired at a low cost, such as by monitoring the easily noticeable singing behavior of other individuals [7]. Therefore, conspecific cues are a valuable type of social information about habitat, and many bird species have been shown to rely on these cues in breeding-site selection (reviewed in [7], [8]).

When using conspecific cues, birds follow a conspecific attraction strategy and prefer to settle in patches already occupied by other individuals, which leads to locally aggregated distributions, especially for songbirds that usually do not maintain exceptionally large territories [2], [3], [8]. Although this strategy of making settlement decisions is most likely widespread among songbirds, the fitness consequences for individuals are largely unexplored [7]. Using social information is considered a less time- and energy-consuming strategy of assessing habitat quality than the direct sampling of various habitat patches [9–11]. However, non-selective copying of the decisions made by others may lead to maladaptive choices and settlement in poor habitats [12], [13]. For example, Nocera et al. [14] showed that natal dispersing Bobolinks (*Dolichonyx oryzivorus*) blindly relied on simulated social cues for breeding site selection and settled in poor-quality habitats where these birds normally do not occur. Therefore, certain costs and benefits of using social information are directly related to the process of reducing individual uncertainty; however, the clustered distribution that emerges as a by-product of conspecific cues may be associated with several predation-related costs and benefits that might influence the fitness of individuals [7], [8].

A benefit of conspecific attraction that is often assumed to be related to predation is protection from predators through the dilution effect of clustered breeding [2], [8], [15]. According to this hypothesis, the larger the group of individuals, the lower the risk that a given nest will be depredated. Therefore, following a conspecific attraction strategy may potentially provide an additional benefit, as birds with clustered territories may reach higher nest success levels than those in more dispersed territories [2], [8]. However, territory clusters may facilitate nest localization if predators respond functionally to prey distribution, e.g., by performing a more restricted search of nearby areas after encountering a nest [16–18]. If predators perform such an adaptive switch in foraging behavior, this may lead to greater predation risk within aggregations and potentially result in a fitness cost for prey. Although the dilution effect is expected to provide benefits for clustered birds in a landscape dominated by incidental nest predators, nesting in the aggregation may be risky in the presence of predators that perform area-restricted searches [19], [20]. Therefore, we hypothesized that the fitness costs and benefits of following a conspecific attraction strategy could largely depend on the composition of the predator community.

Individuals are expected to adjust breeding-site selection according to information that allows them to assess environmental conditions more reliably [21]. Songbirds have been shown to perceive information related to risks posed by different predator types and use this information in the habitat selection process (e.g., [22–24]). Therefore, clustered individuals may attempt to increase their chances for successful breeding by perceiving the risk associated with clustering and adjusting settlement decisions accordingly. Although birds may initially select breeding habitats based solely on conspecific cues, they may avoid nesting in the close proximity of neighbors if the number of newly arriving birds at a particular location exceeds some threshold value. Therefore, individuals may use Bayesian updating of prior settlement decisions by combining different kinds of information about various environmental parameters to improve decision-making processes [25], [26]. This strategy leads to nesting in looser clusters and may be profitable for both birds that rely on conspecific cues (cue-users) and individuals that are social cues for others (cue-providers). Many bird species have been shown to use Bayesian updating in food patch exploitation (reviewed in [25]). However, because the economy of using social information in a habitat-selection process is a largely unexplored phenomenon [7], [13], the potential advantages of using Bayesian updating when making settlement decisions remain unknown.

Knowledge of the fitness consequences of using conspecific cues constitutes one of several urgent research needs [7] that is not purely academic because the manipulation of social information is often considered as a potential tool in the conservation of songbird populations [5], [8], [27]. Although there is an apparent lack of empirical data from field experiments, an agent-based approach provides an opportunity to investigate these issues. We developed an individual-based model to explore the predation-related costs and benefits of following a conspecific attraction strategy in a multi-predator landscape. The model was designed to simulate the nest success of the Wood Warbler (*Phylloscopus sibilatrix*), a small, forest-dwelling songbird. Specifically, we aimed to examine the following: (1) the fitness consequences of following a conspecific attraction strategy in landscapes dominated by different predator types and (2) whether Bayesian updating of prior settlement decisions may improve the fitness of birds that rely on conspecific cues.

## Methods

### Model setup

The model was created using the open-source software NetLogo ver. 5.0.4 [28]. All empirical values for model parameterization were obtained from the existing literature and our unpublished results obtained from Wood Warbler studies in the Wielkopolska National Park (western Poland). Unpublished data that were used are included as Supplementary Material (S1 File). Below, we briefly describe the basic model features: the simulation space, agents and model implementation. The model and full ODD protocol [29], [30] are provided as Supplementary Material (S2 File, S3 File).

The simulation space was designed to represent one of the study plots (Wiry Forest) in our study area—the Wielkopolska National Park. It was a discrete 2D square grid of 200 × 200 cells. Each cell represented an empirical distance of 10 m. Hence, the modeled space covered an area of 4 km^2^, which is approximately equal to the area of the Wiry Forest (4.65 km^2^). The simulation space did not contain obstacles or artificial boundaries and was modeled as a torus.

Each run of this model consisted of two stages: the simulation of birds’ settling and the foraging behavior of predators. We simulated two types of predators i.e., Wild Boar (*Sus scrofa*) and Red Fox (*Vulpes vulpes*), which differed in their foraging behavior. During the first stage, time was not specified (see S3 File for details). The simulation time of the second stage was scaled based on data regarding Red Fox movement during foraging bouts [20], [31]. Time moved forward in discrete steps of fixed size, i.e., 1 tick per step in all simulations. We considered the following: (1) predators moved forward 1 grid cell per time step; (2) in nature, foxes often move ~2 km per foraging bout [31]; and (3) a single cell represented an empirical distance of 10 m; we defined one day as 200 time steps. Because the nest cycle of Wood Warblers lasts 32 days, all simulations were run for 6400 time steps. We assumed constant predation risk in each model run.

### Bird and nest agents

The model included three sub-types of bird agents that exhibited a different settling strategy: cue-providers, cue-users and random-settlers.

The cue-providers represented birds that made settlement decisions based on personal information. During each model initialization, the cue-providers were distributed randomly within a simulation space at least 10 cells away from the nearest neighbor because the mean nearest-neighbor distance between territories of Wood Warblers in the Wiry Forest (Wielkopolska National Park) equals 102.21 m (n = 92 pairs) (Szymkowiak and Kuczyński, S1 File).

The cue-users represented birds that relied on social information, i.e., birds that made settlement decisions based on the location of other individuals who had already occupied territories. During each model initialization, the cue-users were distributed randomly within a simulation space but were at least 10 cells away from the nearest cue-provider. After all cue-users were placed in the modeled space, each of them started prospecting behavior, during which they moved in a correlated random walk (independent from each other) and became territorial after acquiring social cues. The correlated random walk is a practicable approach to describing animal movement patterns in which an individual’s trajectory through space is regarded as a sequence of distinct and randomly oriented steps, with the direction of a single step related to the direction of a previous step [32].

The random-settlers represented birds that settled at random, i.e., birds that did not rely on any specific type of information when making settlement decisions. After both other types of bird agents became territorial, the random-settlers were placed within a simulation space. Although they represent randomly settled individuals, random-settlers were distributed under the following two restrictions: individuals were least 10 cells away from other random-settlers and at least 20 cells away from the nearest cluster of cue-providers and cue-users. The first restriction resulted from the average nearest-neighbor distance between Wood Warbler territories in the Wiry Forest (Wielkopolska National Park). The second limitation had to be introduced because simulations included all bird agent types. Hence, there was a risk that random-settlers in a purely random distribution would be located in the middle of a group of clustered birds (i.e., cue-providers and cue-users), which might influence the results to a great extent and lead to misleading conclusions. It is also important to note that from a modeling perspective, both cue-providers and random-settlers were initially distributed similarly within a simulation space. However, in a biological sense, we implicitly assumed that cue-providers were birds that relied on personal information, such as their own breeding experience from the previous year, whereas random settlers made uninformed decisions. Moreover, both agent types could be distinguished because cue-providers were involved in social interactions with cue-users, which was not the case for random-settlers.

The nests were stationary agents and were distributed randomly within the bird agent’s territories. Although each bird agent occupied a territory with a radius of 5 cells (representing an empirical distance of 50 m), nests were sprouted randomly within a radius of 3 cells measured from the territory center. This approach was intended to account for the mean empirical distance between the territory center and nest location, which equals 32.45 m (n = 47) in our study plot (Szymkowiak and Kuczyński, S1 File).

### Conspecific attraction behavior

Two types of conspecific attraction strategies were implemented, and all cue-users followed one of the strategies in a particular simulation run. Therefore, separate sets of simulations were performed for exploring the costs and benefits of each strategy.

### Basic conspecific attraction

In the basic conspecific attraction strategy, the cue-users moved in a correlated random walk, one cell per time step. The movement heading was drawn at each time step from a normal distribution with a mean of 0° and a standard deviation defined by the observer (see S3 File, A.3.3.1. for details about choosing SD values for cue-user prospecting behavior). At each time step, the non-territorial cue-users were asked whether there was another bird agent in a radius of 10 cells that had already occupied the territory. If another agent was present, the cue-user perceived this information as a social cue, stopped and became territorial (note that after this it could be perceived as a social cue for others); if there was no such individual, the cue-user continued prospecting behavior. This procedure was completed after all territories had been occupied by cue-users.

### Conspecific attraction with Bayesian updating

In this strategy, the cue-users performed a more sophisticated conspecific attraction strategy that included a form of Bayesian updating of prior settlement decisions. First, each cue-user followed a basic conspecific attraction strategy. However, at each time step, all cue-providers and those cue-users that acquired social cues (and were settled) were asked how many other individuals were in a radius of 15 cells. If the number exceeded a threshold value defined by the observer (*k* parameter), bird agents moved (one cell per time step) according to a correlated random walk with a heading drawn from a normal distribution (mean = 0°, SD = 360°). Because the turning angle was high, this process resulted in a very tight, convoluted movement that was continued until the number of individuals in a radius of 15 cells was equal to or lower than the threshold value. If this assumption was fulfilled, the bird agent stopped immediately. Because the birds were asked to assess the number of other territorial individuals in a 15-cell buffer at each time step, this “updating of territory borders” could be induced repeatedly if there were newly arriving birds at a particular location. In some scenarios, however, newly arriving birds could potentially displace individuals that have already occupied territories, which is unlikely from an empirical perspective but was allowed for coding simplicity because it did not affect the results of our analysis. The “updating territory borders” procedure was completed after all cue-providers and cue-users occupied stable territories. In a biological sense, we modeled settlement behavior in which the cue-users followed a conspecific attraction strategy; however, together with the cue-providers, they also perceived the risk associated with clustering and performed Bayesian updating of prior settlement decisions. This strategy is based on the implicit assumption that songbirds are able to adjust site selection decisions when information allowing them to assess habitat quality more reliably becomes available [21]. Such behavior is supported by abundant empirical data demonstrating habitat selection as a dynamic decision-making process during which initial settlement locations could be shifted when more timely information emerges or ecological conditions change (e.g., [33], [34])

### Predator agents

Two types of predator agents were simulated in this model, Wild Boar and Red Fox, which differed in their foraging behavior. Both species are common in our study area and are well known predators of Wood Warbler nests [35], [36]. All predator agents were randomly distributed within a simulation space at the beginning of each model run and were able to eat nests if they occupied the same cell. In each model run, predators could consume an infinite number of nests at any time because we assumed that it would be unlikely for a boar or fox to be satiated after consuming a typical Wood Warbler clutch containing 6 small eggs [35].

The Wild Boar represented an incidental predator of Wood Warbler nests. The boars moved forward in a correlated random walk, one grid cell per time step. The direction of movement was drawn at each time step from a normal distribution with a mean of 0° and standard deviation defined by the observer (*turning*.*boar* parameter). All standard deviations for predator movement parameters used in the final experiments were chosen based on sensitivity analyses and made constant across the simulations (see Efficient predator setup for details). In nature, Wild Boars and Red Foxes move different distances during foraging bouts; whereas foxes move ~2 km daily [20], [31], boars are expected to move ~1 km daily [37]. The time of the entire simulation was scaled based on data for the average distance moved by Red Foxes during foraging bouts. Hence, to account for differences in the activity patterns of both predators, boar activity was constrained to 100 time steps per day (see *A*.*3*.*3*.*4*. in S3 File for details).

The Red Fox represented a predator with a capacity to perform more intensive searching of nearby areas after encountering a nest. Normally, foxes moved in a correlated random walk with a movement heading that was drawn, at each time step, from a normal distribution with a mean of 0° and a standard deviation defined by the observer (*turning*.*fox* parameter). However, foxes changed their movement strategy and performed area-restricted searches (ARSs) after encountering a nest. During the ARS movements, the foxes’ turning angle increased (*strength*.*ARS* parameter), and they explored nearby areas instead of moving further away. The duration of ARS movements was controlled by the observer-defined parameter (*duration*.*ARS* parameter). If the fox agent did not find any other nests during this time, it resumed movement in a standard correlated random walk. The ARS foraging strategy is based on the implicit assumption made by a predator about the higher probability of encountering other food resources near a previously located nest. As noted by Ringelman [20], this movement strategy is qualitatively similar to Lévy flight behavior, i.e., random walks in which the short movement bouts are interspersed with long movements [38]; foxes have been shown to follow this strategy when searching for prey [20], [31], [39].

### Model initialization

The model was initialized with 80 total bird agents (and their nests), which is equivalent to the density of breeding Wood Warbler pairs in the Wiry Forest of the Wielkopolska National Park. Wood Warblers exhibit little site tenacity, with a median of 11% of males returning to previous breeding areas (n = 4 publications, based on Table 1 in [40]). We assumed that these males rely on personal information when making settlement decisions; thus, 8 cue-providers were initially placed in the simulation space. Among the remaining 72 bird agents, 36 were modeled as cue-users and 36 as random-settlers.

**Table 1:** Parameter values (on the logit scale) of the model relating nest success when foxes forage with the threshold value that initiated Bayesian updating of prior settlement decisions.

	Mean	SE	2.5% CI	97.5% CI
alpha	-0.49149	0.03103	-0.55109	-0.42941
gamma[k = 2]	0.10047	0.04183	0.01822	0.18327
gamma[k = 3]	0.14617	0.04322	0.06081	0.22892
gamma[k = 4]	0.09886	0.04578	0.00651	0.18687
gamma[k = 5]	0.04388	0.04559	-0.04715	0.13150
gamma[k = 6]	0.03066	0.04452	-0.05609	0.11892

Estimated posterior means, standard errors and 95% credible intervals are shown. If the *k* parameter was in the range of 2–4, there was a significant advantage of applying Bayesian updating. For *k* > = 5, Bayesian cue-providers and cue-users did not gain additional fitness benefits. Alpha is the overall mean, and gamma is the contrast expressing fitness gains in relation to the baseline level, i.e., the naive strategy.

We are not aware of up-to-date estimates of predator densities at our study site; therefore, we preliminarily explored the model setup to select the optimal number of predators for final simulations. We systematically varied the number of boars and foxes (range: 1–8 individuals, 50 simulations for each combination) and calculated the nest survival of clustered and randomly distributed birds. Simulations were performed separately for each predator type. After preliminary model exploration, we decided to simulate 6 predators that caused the mean nest failures within the observed ranges of Wood Warbler nest success (ca. 20–60%) [35], [36], [41].

### Simulation runs and statistical analysis

First, we systematically varied the parameter space of boar and fox movement behavior, attempting to find a setup that was most efficient from the predator’s perspective, causing the lowest nest survival of the entire bird population. Each parameter setup was modeled in 50 simulations. The optimal foraging strategy could largely depend on nest distribution [20]; thus, we modeled either 80 random-settlers (random distribution) or 8 cue-providers with 72 cue-users performing a basic conspecific attraction strategy (clustered distribution). Then, the efficiency of the predators was explored separately for the two bird communities.

After creating efficient boars and foxes, we performed a set of experiments to explore the predation-related costs and benefits of various settling strategies in landscapes with different predator communities. In each experiment, a mixed bird community was simulated, and the number of each bird agent sub-type was constant between experiments. Then, we compared the fitness costs and benefits of following a particular settling strategy by comparing the nest success of cue-providers, cue-users and random-settlers.

Data were analyzed in a Bayesian approach using a binomial GLM with a logit link function (which is equivalent to a logistic regression). When testing for differences in nest success between birds applying different settling strategies, a simple model contrasting groups was used:

Equation 1:

$$\begin{array}{l} s_{i}=Bin\left(N_{i},p_{i}\right)\\ \mathrm{logit}\left(p_{i}\right)=\alpha_{j\left(i\right)} \end{array}$$

where *p_i_* is the probability of success for the *i-*th simulation, *s_i_* is the number of successful nests for the *i-*th simulation, *α_j(i)_* is the logit-scale parameter for expected probability of success for birds applying strategy *j*, and *N_i_* is the known overall number of nests involved in each simulation. This approach is equivalent to a means parameterization in binomial GLMs [42].

This model was slightly modified when testing for differences between strategies in a multi-predator environment:

Equation 2:

$$\begin{array}{l} s_{i}=Bin\left(N_{i},p_{i}\right)\\ \mathrm{logit}\left(p_{i}\right)=\alpha_{j\left(i\right)}+\beta_{j\left(i\right)}\times Nfoxes_{i} \end{array}$$

where *Nfoxes_i_* is the number of foxes in a predator community for the *i-*th simulation, *β_j(i)_* is the logit-scale parameter for slopes for the relationship between number of foxes and nest success for birds applying strategy *j*, and the other parameters were the same as in the previous model. This parameterization is equivalent to a binomial ANCOVA [42].

For testing whether Bayesian birds achieved any fitness gain and how this was related to the number of individuals in a close neighborhood that initiated the updating of prior settlement decisions, the following model was used:

Equation 3:

$$\begin{array}{l} s_{i}=Bin\left(N_{i},p_{i}\right)\\ \mathrm{logit}\left(p_{i}\right)=\alpha +\beta_{j\left(i\right)}+\gamma_{k\left(i\right)} \end{array}$$

where *α* is an overall mean nest success (on logit-scale), *β_j(i)_* is the logit-scale parameter for expected probability of success for birds applying strategy *j* and *γ_k(i)_* is the logit-scale parameter for the expected probability of success for birds applying Bayesian updating when the local density is higher than *k*. The value of *k = 0* was assigned to the naive strategy, i.e., to birds that did not perform updating of settlement decisions. To make the model identifiable, priors of the first levels of both predictors were set to zero, which is equivalent to the effects parameterization in GLMs [42]. Thus, the estimated parameters provide contrast and express differences between the group means and appropriate reference levels (cue-providers in the case of *β* and naive birds in the case of *γ*).

Statistical analyses were performed using JAGS Gibbs-sampling environment [43] and R 3.0.3 [44]. Both environments were integrated via the R2Jags library [45]. Differences in parameters (e.g., group probabilities, slopes) were tested by computing the contrasts (reciprocal cross differences) within JAGS and checking whether the 95% credible intervals (CI thereafter) of those contrasts contained zero. Vague normal density priors were used for all covariate parameters. Gibbs sampling was performed with three independent chains for 10000 iterations each. The first 4000 iterations of each chain were discarded as burn-in, and only samples from every 10th iteration were stored. The JAGS code is provided as Supplementary Material (S4 File).

## Results

### Efficient predator setup

First, we varied the parameters that determined the straightness of predator movement. The nest survival increased when predator paths became more convoluted and reached a minimum value when the *turning* parameter equaled 10°, irrespective of the nest distribution. Then, we set *turning*.*fox* = 10° and *duration*.*ARS* = 125 time steps and systematically varied the *strength*.*ARS* parameter. We found that predators caused the lowest nest survival when *strength*.*ARS* was between 40 and 70° for both clustered and randomly distributed nests. Next, we set *turning*.*fox* = 10° and *strength*.*ARS* = 55° and systematically varied the *duration*.*ARS* parameter. The results revealed that the survival of randomly distributed nests increased when foxes spent more time performing area-restricted searches, whereas for clustered nests, this relationship was reversed. A reasonable trade-off was chosen, and we set a *duration*.*ARS* = 100 time steps for further analyses. Overall, our sensitivity analysis revealed that the parameter values for efficient predator setup were similar to those in Ringelman’s model [20].

### Conspecific attraction costs and benefits

**Fig 1:**
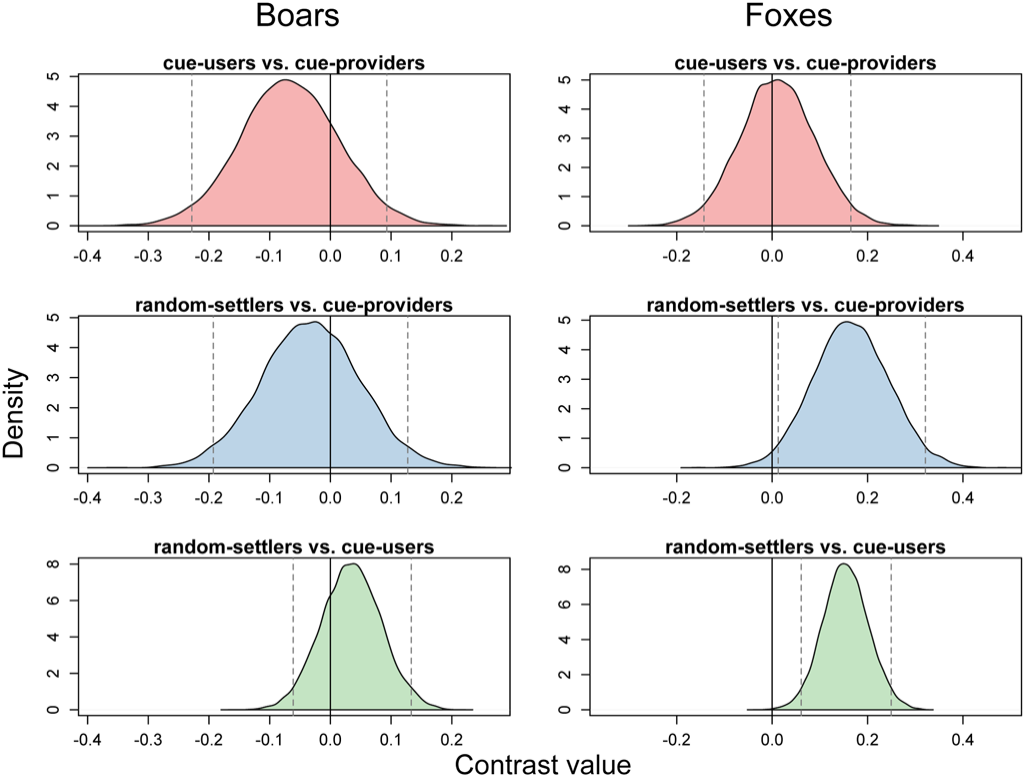
Posterior distributions of different of nest success probabilities of birds following particular settling strategies. The left panel refers to a landscape dominated by boars, and the right panel refers to a fox-only environment. Vertical solid lines indicate zero, and dashed lines indicate 95% credible intervals around the mean difference. In a landscape dominated by incidental predators (boars), there were no significant differences (credible intervals always contain zero), meaning that the three strategies performed equally well. When predators were able to perform area-restricted search (foxes), a random settling strategy significantly outperformed both social strategies, whereas there were no significant differences in nest success between cue-users and cue-providers.

In the first experiment (100 simulations), 6 Wild Boars were simulated to test whether the cue-providers and cue-users benefited from clustered distribution in a landscape dominated by incidental predators. We found no differences in nest success between birds involved in a conspecific attraction mechanism (cue-providers: 64.6%, CI: 61.3–67.9; cue-users: 63.1%, CI: 61.5–64.6). Moreover, the nest success of random-settlers (63.9%, CI: 62.3–65.4) was similar and did not differ from any of the “social” groups (Fig. 1).

In the subsequent experiment (100 simulations), 6 Red Foxes were modeled to test whether clustering as a by-product of conspecific attraction resulted in fitness costs in a landscape dominated by predators that were capable of performing area-restricted searches. The birds that settled at random performed better, i.e., had a higher nest success (posterior mean: 43.6%, CI: 42.0–45.2), than those with clustered territories (cue-providers: 39.6%, CI: 36.3–43.1; cue-users: 39.8%, CI: 38.3–41.5). Furthermore, as indicated by the comparison of posterior distributions of cross-differences (Fig. 1), the nests of clustered birds had similar survival rates; however, the nest success for both the cue-providers and cue-users differed from the random-settlers.

In the next experiment, we examined the fitness costs of random-settling and conspecific attraction in a multi-predator landscape. We systematically varied the numbers of boars and foxes (range: 0–6 individuals, 100 simulations for each combination) to simulate a mixed predator community. However, the total number of predators in each set of simulations was held constant at 6. Although we included only the number of foxes (*N*.*foxes*) as a predictor in the model, this represented a gradient between landscapes dominated by incidental predators (boars) and those dominated by predators that performed area-restricted searches (foxes).

**Fig 2:**
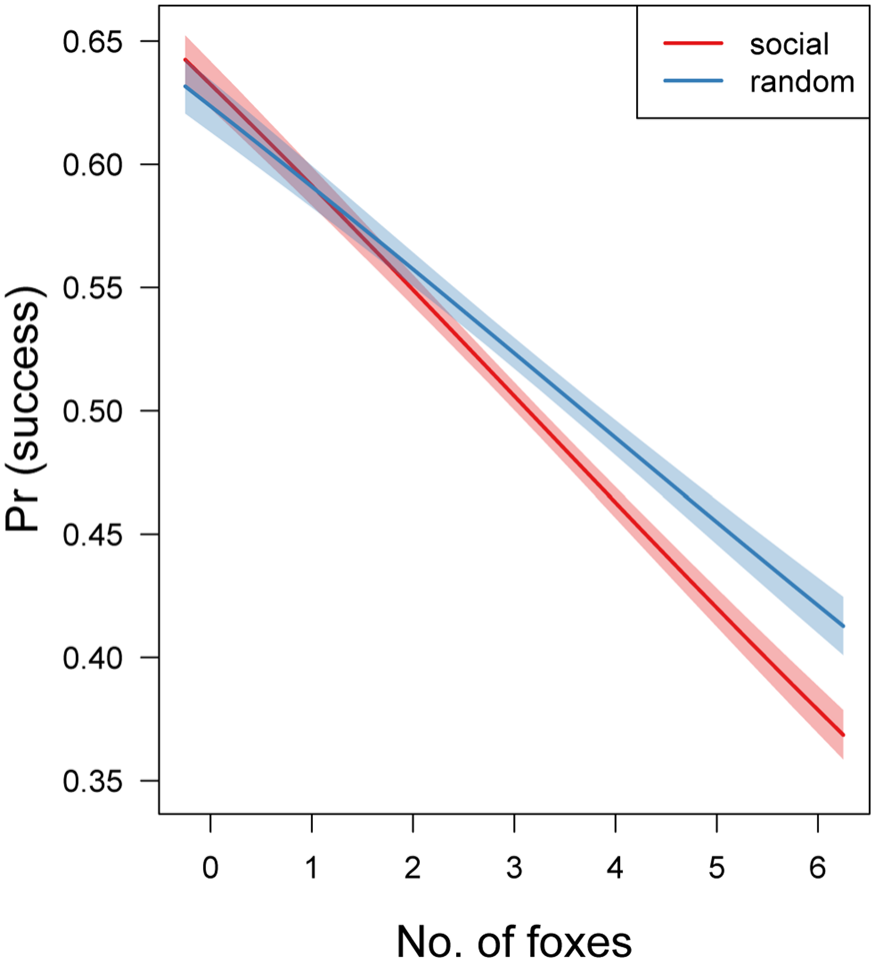
Probability of nest success in a multi-predator landscape varying in domination of foxes. Fitted lines and their 95% credible intervals for both “social” (red) and “random” (blue) settlers are shown. The random settling strategy became superior when foxes constituted at least half of the predator community.

The binomial ANCOVA model fitted using MCMC revealed that the mean nest success for the three settling strategies was similar and did not differ (posterior mean intercepts: 54.5%, CI: 44.8–64.4; 54.2%, CI: 49.7–58.9; 50.6%, CI: 46.1–55.2, for cue-providers, cue-users and random-settlers, respectively). However, the comparison of slopes and their reciprocal contrasts showed that although they were not different (at the 95% CI level) between the cue-providers and cue-users, both differed from the random-settlers. Thus, the model was refitted with both cue-providers and cue-users pooled together into a “social” group. Similar conclusions can be inferred from this simplified model: intercepts did not differ (“social”: 54.2%, CI: 50.0–58.4 vs. random-settlers: 50.5%, CI: 46.0–55.1), but the slopes did differ (“social”: -0.173, CI: -0.185–0.162 vs. random-settlers: -0.137, CI: -0.150–0.125). The posterior distribution of the contrast between both estimated slopes did not cover zero, which indicates that the nest survival of random-settlers did not decrease as rapidly as for birds with clustered territories (Fig. 2).

### Profitability of Bayesian updating

In the final experiments, we examined whether acquiring information about the social environment and performing Bayesian updating of prior settlement decisions could lead to fitness benefits for birds that bred in clusters as a result of conspecific attraction. We systematically varied the *k* parameter (range: 2–6, 100 simulations for each value) and modeled the nest success of Bayesian cue-providers and cue-users in a landscape of “pure” predator communities with either 6 boars or 6 foxes. Moreover, in a separate set of simulations (100 each time), we modeled the nest success of naive cue-providers and cue-users and then compared it with that obtained by Bayesian birds at different *k* values.

The first model considered was a two-way binomial ANOVA with two factors: settling strategy and *k* parameter. For both boar- and fox-only environments, the cue-providers and cue-users performed equally well in this setup, and there were no differences between them. Thus, the model was refitted with only one predictor (*k*).

**Fig 3:**
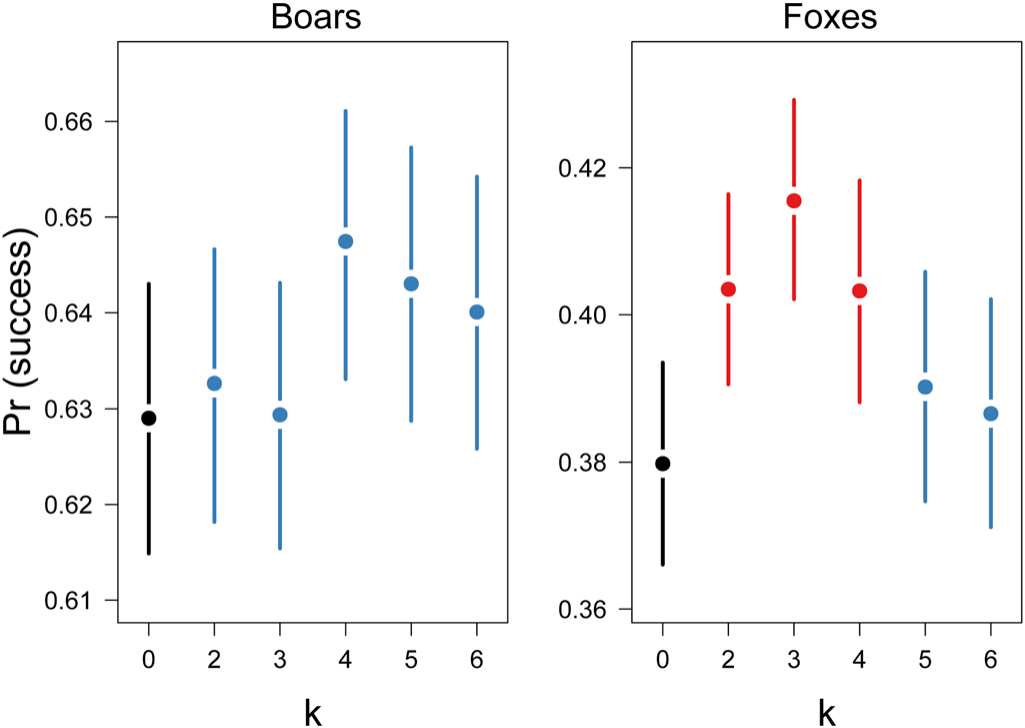
Fitted nest success probabilities plotted against the *k* parameter for boars-only (left panel) and fox-only (right panel) environments. Points represent posterior means, and lines represent 95% credible intervals. The reference value for k = 0 (in black) denotes the mean success for naive birds which did not perform Bayesian updating of prior settlement decisions. Effects that are not different from the reference are in blue, whereas different effects are shown in red.

When the landscape was dominated by predators able to perform area-restricted searches, it was profitable for birds to use Bayesian updating of prior settlement decisions. Bayesian cue-providers and cue-users performed better (i.e., reached a higher nest success) than naive birds when the *k* parameter was 2, 3 or 4; however, when the value was ≥ 5, the fitness benefits of Bayesian updating disappeared (Fig. 3, Table 1).

As expected, using a Bayesian approach in the landscape dominated by incidental predators did not provide any benefits. We did not find any relationship between the fitness of birds involved in a conspecific attraction mechanism and the *k* parameter. Moreover, the nest success of naive cue-providers and cue-users did not differ from those achieved by individuals that performed Bayesian updating (Fig. 3, Table 2).

**Table 2:** Parameter values (on the logit scale) of the model relating nest success when boars forage with the threshold value that initiated Bayesian updating of prior settlement decisions.

	mean	SE	2.5% CI	97.5% CI
alpha	0.53115	0.03139	0.47039	0.59325
gamma[k = 2]	0.01216	0.04490	-0.07667	0.10042
gamma[k = 3]	-0.00046	0.04414	-0.08716	0.08335
gamma[k = 4]	0.07651	0.04493	-0.01272	0.16497
gamma[k = 5]	0.05648	0.04446	-0.03031	0.14279
gamma[k = 6]	0.04377	0.04373	-0.04206	0.13015

Estimated posterior means, standard errors and 95% credible intervals are shown. Bayesian cue-providers and cue-users did not gain additional fitness benefits compared with naive birds in relation to nest success. Alpha is the overall mean, and gamma is the contrast expressing fitness gains in relation to the baseline level, i.e., the naive strategy.

## Discussion

Conspecific attraction alone is beneficial for birds, as it is a way of gathering habitat information and making informed settlement decisions that allow for better adjustment to current environmental conditions [7], [8], [46], [47]. However, our results provide strong evidence that there are also fitness costs of following a conspecific attraction strategy. Moreover, this trade-off depends on the composition of the predator community.

Apart from reducing the uncertainty of individuals, studies have suggested that clusters of territories that emerge as a by-product of conspecific attraction may provide benefits through the dilution effect [2], [8], [7], [15]. This phenomenon should particularly be expected in a landscape with incidental predators that typically forage on alternative prey species [19], [20]. However, our results did not support this hypothesis. When we simulated the foraging behavior of Wild Boar, the nest success of clustered and randomly distributed nests did not differ. Therefore, the cue-providers and cue-users did not gain any additional fitness benefits from nesting within an aggregation, as would be expected according to the dilution effect hypothesis. Recently, Ringelman [20] used an agent-based approach to simulate predator foraging behavior qualitatively similar to that presented in this model, with results differing from those of this study: a better survival of clustered nests was found in the presence of incidental predators. One possible explanation for these inconsistencies is the different number of nests that incidental predators could consume. In Ringelman’s model [20], predators were satiated for the day after consuming a nest, whereas in our model, boars could “consume” an infinite number of nests at any time. However, we suggest that a more plausible explanation for inconsistent results between both studies is related to the different features of the natural systems that were modeled. Ringelman [20] simulated the nest survival of tightly clustered dabbling ducks, whereas we modeled the settling behavior of the Wood Warbler, which is a species that occupies inherently larger all-purpose territories [48]. This difference resulted in substantially larger nearest-neighbor distances and less tight clustering in warblers than observed in ducks [49], [50]. It seems intuitive that when the distance between neighboring nests increases, the benefits from dilution effects disappear, even if territories are still spatially aggregated. In fact, Ringelman [20] noted that although clustered nests survived better than random nests in the presence of incidental predators, the relative benefits from clustering decreased when nest dispersion increased. However, most of the songbirds that have been shown to rely on conspecific cues occupy all-purpose territories [7]. Therefore, the often-suggested benefit of clustering through conspecific attraction, i.e., the dilution effect, may not occur in these species and should be verified with field studies addressing the spatial scale of the aggregative behavior exhibited by songbirds.

Because we modeled the settling behavior of birds with all-purpose territories, which resulted in a looser distribution of nests than for ducks (although still spatially aggregated), one could expect that the profitability of area-restricted searches might disappear from the predator’s perspective [20], [39]. Instead, we found that predators were still more efficient when changing their foraging behavior after encountering a nest. Although the nests of all bird agents had lower survival rates when foxes became the dominant predators, which was most likely caused by differences in the activity patterns of the predators, with foxes foraging twice as long as boars, the nest success of clustered birds was reduced significantly more than that of random-settlers. Therefore, in the presence of foxes, both the cue-providers and cue-users were faced with fitness costs that resulted from the clustered distribution. The game-theoretic model of Doligez et al. [10] suggested that various strategies of habitat selection performed poorly when individuals tended to aggregate in space. This effect was due to the density-dependent processes that induced costs in the form of reduced fecundity. It appears that there are also other costs of these settling strategies, including conspecific attraction, that emerge when predators respond behaviorally to prey distribution, thus leading to density-dependent nest predation [17].

The costs of clustering may decrease when birds perceive that there is a risk of nesting with too many neighbors and perform Bayesian updating to adjust prior settlement decisions accordingly. However, the profitability of this strategy vanishes when birds accept to settle in clusters with high number of conspecifics. In our model, the cue-providers and cue-users followed simple rules: they moved a small distance away from each other if the number of neighbors exceeded a threshold-value. This behavior showed that Bayesian birds tended to nest in clusters with fewer neighbors and had territories that were less tightly spaced, which ultimately spread the associated risks. Thus, Bayesian updating was expected to be profitable in landscapes dominated by predators able to perform area-restricted searches [20]. In fact, we found that Bayesian cue-providers and cue-users achieved a higher nest success than naive birds in the presence of foxes. Therefore, perceiving the risk associated with clustering and adjusting settlement decisions accordingly may improve the fitness of individuals and may thus be adaptive. It is important to note that the actual model code did not completely preclude the scenario in which two birds randomly moved simultaneously without increasing their distance from each other for a certain time period; however, this behavior has the potential to spread the cluster farther, which might amplify these results (see *A*.*3*.*3*.*2*. in S3 File). Nevertheless, the simple Bayesian updating strategy presented here appears to be parsimonious and easy to apply by clustered individuals. Currently, there is a growing body of evidence that birds are able to perceive information about the risks posed by different predator types and use it in a habitat selection process (e.g., [22–24]). Moreover, many animals have been shown to behave as Bayesians in various contexts such as foraging and mate choice [25], [51]. It is unknown, however, how birds update prior decisions and combine different types of information in the habitat selection process [7]. Although these phenomena can be difficult to study empirically using controlled field experiments, an agent-based approach allowed us to explore them.

Conspecific attraction is a form of intraspecific interaction between cue-users and cue-providers that is likely to be widespread among songbirds [7], [8]. To date, however, no empirical studies have been performed that would evaluate whether this interaction is parasitic (cue-user gains benefits at the cue-provider’s expense), commensal (i.e., neutral for cue-providers) or mutualistic (beneficial for both cue-users and cue-providers) (see [52] for a heterospecific attraction example). Our results suggest that in a landscape dominated by foxes, the cue-providers incurred the cost of social cues because the probability of successful breeding decreased when cue-users settled near them. This cost, however, vanished when the predator community was dominated by incidental predators. This finding suggests that for cue-providers, the outcome of the interaction with cue-users might be locally specific and depend on the local environmental conditions [7]. However, the exact evaluation of the interaction between cue-providers and cue-users is difficult based solely on our model because the overall fitness consequences of conspecific attraction for cue-users cannot be fully determined. The reliance on conspecific cues allows cue-users to make rapid habitat quality assessments when deciding where to settle, even when they lack prior knowledge about a given area [7], [8], [53]. When patch quality is spatially diverse (not analyzed here), random settling is not an efficient strategy and leads to maladaptive habitat choices; therefore, acquiring information about the environment improves fitness [7], [10]. It might be beneficial to introduce habitat heterogeneity in future models to more precisely quantify the fitness consequences of conspecific attraction for cue-users, thereby allowing for more accurate evaluations of the outcome of cue-provider and cue-user interactions.

We did not observe any significant differences between the nest success of cue-providers and cue-users in the landscapes dominated by both foxes and boars. The same patterns were found for birds that performed Bayesian updating behavior. This supports the hypothesis that individuals within clusters formed as a by-product of conspecific attraction nested under similar predation risks [7]. However, we did not analyze nest success in relation to the spatial position of nests within clusters. Several studies have suggested that within aggregations, internal nests are less vulnerable to depredation than marginal nests (selfish herd effect hypothesis) [54–56]. If cue-users aggregate territories around cue-providers, which is similar to the “hotshot model” of hidden lek evolution [57], and predation risk is disproportionally allocated within clusters, then the fitness of cue-providers will be favored. However, we modeled a more parsimonious attraction mechanism in which the birds made settlement decisions based on the settling status of other individuals. When the simulation began, only the cue-providers that relied on personal information were territorial; cue-users became territorial after acquiring social information and thereafter could be perceived as providing social cues for other individuals. Therefore, prospecting cue-users had the ability to respond to both cue-providers and recently-settled cue-users with increased simulation time. Thus, initial cue-providers were not always settled at the geometric center of the cluster, preventing us from testing the hypothesis that fitness benefits caused by predation risk were disproportionally allocated within clusters. Further model developments should track nest survival with respect to their spatial position within clusters, in addition to modeling different scenarios of bird attraction.

The manipulation of social cues has often been considered as a potential method in the conservation and management of songbird populations (e.g., [5], [8], [26], [58], [59]). However, the results from our model suggest that there may be a risk of using artificial-attraction methods in conservation if birds use conspecific cues non-selectively and blindly copy the decisions made by other individuals. When the landscape is dominated by predators able to perform area-restricted searches, the clustering distribution did not perform well and led to lower nest survival. Therefore, manipulating conspecific cues in such environments may create ecological traps, as individuals will prefer to settle in conditions that adversely affect their fitness [7], [60], [61]. Similar concerns were expressed by Ringelman [20] in the context of management strategies that force ducks to nest in clusters without being aware of the predation-related costs of such practices. Therefore, we suggest that when considering artificial attraction as a method in the conservation of songbird populations, managers should consider the composition of the local predator community to avoid the risk of creating ecological traps.

## Supporting Information

S1 File Unpublished data on the nearest-neighbour distance between territories of Wood Warblers in the Wiry Forest and the distance between the nest location and territory center. (XLSX)

S2 File The NetLogo file of the model. (NLOGO)

S3 File The ODD model description. (DOCX)

S4 File The JAGS code used in statistical analyses. (7Z)

## References

[1] Alatalo RV, Lundberg A, Björklund M. Can the song of male birds attract other males? An experiment with the Pied Flycatcher *Ficedula hypoleuca* . Bird Behavior. 1982; 4: 42–45.

[2] Stamps JA. Conspecific attraction and aggregation in territorial species. Am Nat. 1988; 131: 329–347.

[3] Stamps JA. The effects of conspecifics on habitat selection in territorial species. Behav Ecol Sociobiol. 1991; 28: 29–36.

[4] Danchin É, Giraldeau LA, Valone TJ, Wagner RH. Public information: from nosy neighbors to cultural evolution. Science. 2004; 305: 487–491. 15273386 10.1126/science.1098254

[5] Ward MP, Schlossberg S. Conspecific attraction and the conservation of territorial songbirds. Conserv Biol. 2004; 18: 519–525.

[6] Fuller R. Birds and habitat: relationships in changing landscapes. Cambridge: Cambridge University Press; 2012.

[7] Szymkowiak J. Facing uncertainty: how small songbirds acquire and use social information in habitat selection process? Springer Science Reviews. 2013; 1: 115–131.

[8] Ahlering MA, Arlt D, Betts MG, Fletcher RJ, Nocera JJ, Ward MP. Research needs and recommendations for the use of conspecific-attraction methods in the conservation of migratory songbirds. Condor. 2010; 112: 252–264.

[9] Mönkkönen M, Forsman JT. Heterospecific attraction among forest birds: a review. Ornithol Sci. 2002; 1: 41–51.

[10] Doligez B, Cadet C, Danchin É, Boulinier T. When to use public information for breeding habitat selection? The role of environmental predictability and density dependence. Anim Behav. 2003; 66: 973–988.

[11] Seppänen JT, Forsman JT, Mönkkönen M, Thomson RL. Social information use is a process across time, space, and ecology, reaching heterospecifics. Ecology. 2007; 88: 1622–1633. 17645008 10.1890/06-1757.1

[12] Giraldeau LA, Valone TJ, Templeton JJ. Potential disadvantages of using socially acquired information. Philos T R Soc B. 2002; 357: 1559–1566. 12495513 10.1098/rstb.2002.1065 PMC1693065

[13] Rieucau G, Giraldeau LA. Exploring the costs and benefits of social information use: an appraisal of current experimental evidence. Philos T R Soc B. 2011; 366: 949–957. 10.1098/rstb.2010.0325 21357217 PMC3049093

[14] Nocera JJ, Forbes GJ, Giraldeau LA. Inadvertent social information in breeding site selection of natal dispersing birds. Proc R Soc B. 2006; 273: 349–355. 16543178 10.1098/rspb.2005.3318 PMC1560037

[15] Turner GF, Pitcher TJ. Attack abatement: a model for group protection by combined avoidance and dilution. Am Nat. 1986; 128: 228–240.

[16] Krause J, Godin JGJ. Predator preferences for attacking particular prey group sizes: consequences for predator hunting success and prey predation risk. Anim Behav. 1995; 50: 465–473.

[17] Schmidt KA, Whelan CJ. Nest predation on woodland songbirds: when is nest predation density dependent? Oikos. 1999; 87: 65–74.

[18] Ioannou CC, Krause J. Searching for prey: the effects of group size and number. Anim Behav. 2008; 75: 1383–1388.

[19] Andrén H. Predation: an overrated factor for over-dispersion of birds’ nests? Anim Behav. 1991; 41: 1063–1069.

[20] Ringelman KM. Predator foraging behavior and patterns of avian nest success: what can we learn from an agent-based model? Ecol Model. 2014; 272: 141–149.

[21] Dall SRX, Giraldeau LA, Olsson O, McNamara JM, Stephens DW. Information and its use by animals in evolutionary ecology. Trends Ecol Evol. 2005; 20: 187–193. 16701367 10.1016/j.tree.2005.01.010

[22] Thomson RL, Forsman J, Sardà-Palomera F, Mönkkönen M. Fear factor: prey habitat selection and its consequences in a predation risk landscape. Ecography. 2006; 29: 507–514.

[23] Lima SL. Predators and the breeding bird: behavioral and reproductive flexibility under the risk of predation. Biol Rev. 2009; 84: 485–513. 10.1111/j.1469-185X.2009.00085.x 19659887

[24] Morosinotto C, Thomson RL, Korpimäki E. Habitat selection as an antipredator behaviour in a multi-predator landscape: all enemies are not equal. J Anim Ecol. 2010; 79: 327–333. 10.1111/j.1365-2656.2009.01638.x 19912426

[25] Valone TJ. Are animals capable of Bayesian updating? An empirical review. Oikos. 2006; 112: 252–259.

[26] McNamara J, Houston A. The application of statistical decision theory to animal behavior. J Theor Biol. 1980; 85: 673–690. 7442286 10.1016/0022-5193(80)90265-9

[27] Ahlering MA, Faaborg J. Avian habitat management meets conspecific attraction: if you build it, will they come? Auk. 2006; 123: 301–312.

[28] Wilensky U. NetLogo. Center for Connected Learning and Computer-Based Modeling, Northwestern University; 1999. Available: http://ccl.northwestern.edu/netlogo/

[29] Grimm V, Berger U, Bastiansen F, Eliassen S, Ginot V, Giske J, et al A standard protocol for describing individual-based and agent-based models. Ecol Model. 2006; 198: 115–126.

[30] Grimm V, Berger U, DeAngelis DL, Polhill JG, Giske J, Railsback SF. The ODD protocol: A review and first update. Ecol Model. 2010; 221: 2760–2768.

[31] Phillips ML, Clark WR, Nusser SM, Sovada MA, Greenwood RJ. Analysis of predator movement in prairie landscapes with contrasting grassland composition. J Mammal. 2004; 85: 187–195.

[32] Codling EA, Plank MJ, Benhamou S. Random walk models in biology. J R Soc Interface. 2008; 5: 813–834. 10.1098/rsif.2008.0014 18426776 PMC2504494

[33] Betts MG, Rodenhouse NL, Scot Sillett T, Doran PJ, Holmes RT. Dynamic occupancy models reveal within-breeding season movement up a habitat quality gradient by a migratory songbird. Ecography. 2008; 31: 592–600.

[34] Gilroy JJ, Anderson GQA, Grice PV, Vickery JA, Sutherland WJ. Mid-season shifts in the habitat associations of Yellow Wagtails *Motacilla fiava* breeding in arable farmland. Ibis. 2010; 152: 90–104.

[35] Wesołowski T. The breeding ecology of the Wood Warbler *Phylloscopus sibilatrix* in primaeval forest. Ornis Scand. 1985; 16: 49–60.

[36] Mallord JW, Orsman CJ, Cristinacce A, Butcher N, Stowe TJ, Charman EC. Mortality of Wood Warbler *Phylloscopus sibilatrix* nests in Welsh Oakwoods: predation rates and the identification of nest predators using miniature nest cameras. Bird Study. 2012; 59: 286–295.

[37] Diong ChD. Population biology and management of the feral pig (*Sus scrofa*, L.) in Kipahulu Valley, Maui. Ph.D. Thesis, The University of Hawaii. 1982.

[38] Reynolds AM, Rhodes CJ. The Lévy flight paradigm: random search patterns and mechanisms. Ecology. 2009; 90: 877–887. 19449680 10.1890/08-0153.1

[39] Seymour AS, Harris S, White PCL. Potential effects of reserve size on incidental nest predation by red foxes *Vulpes vulpes* . Ecol Model. 2004; 175: 101–114.

[40] Wesołowski T, Rowiński P, Maziarz M. Wood Warbler *Phylloscopus sibilatrix*: a nomadic insectivore in search of safe breeding grounds? Bird Study. 2009; 5: 26–33.

[41] Wesołowski T, Maziarz M. Changes in breeding phenology and performance of Wood Warblers *Phylloscopus sibilatrix* in a primeval forest: a thirty-year perspective. Acta Ornithol. 2009; 44: 69–80.

[42] Kéry M. Introduction to WinBUGS for ecologists: Bayesian approach to regression, ANOVA, mixed models and related analyses. Amsterdam: Elsevier; 2010.

[43] Plummer M. JAGS: A program for analysis of Bayesian graphical models using Gibbs sampling Vienna, Austria: Proceedings of the 3rd International Workshop on Distributed Statistical Computing; 2003.

[44] R Development Core Team. R: A language and environment for statistical computing Vienna, Austria: the R Foundation for Statistical Computing; 2013 Available: http://www.r-project.org/

[45] Su YS, Yajima M. R2jags: a package for running JAGS from R. R package version 0.04–02; 2014. Available: http://cran.r-project.org/web/packages/R2jags/

[46] Dall SRX, Giraldeau LA, Olsson O, McNamara JM, Stephens DW. Information and its use by animals in evolutionary ecology. Trends Ecol Evol. 2005; 20: 187–193. 16701367 10.1016/j.tree.2005.01.010

[47] Schmidt KA, Dall SRX, Van Gils JA. The ecology of information: an overview on the ecological significance of making informed decisions. Oikos. 2010; 119: 304–316.

[48] Glutz von Blotzheim UN, Bauer KM. Handbuch der Vögel Mitteleuropas. Wiesbaden: AULA-Verlag GmbH; 1991

[49] Ringelman KM, Eadie JM, Ackerman JT. Density-dependent nest predation in waterfowl: the relative importance of nest density versus nest dispersion. Oecologia. 2012; 169: 695–702. 10.1007/s00442-011-2228-1 22179311

[50] Ringelman KM, Eadie JM, Ackerman JT. Adaptive nest clustering and density-dependent nest survival in dabbling ducks. Oikos. 2014; 123: 239–247.

[51] McNamara JM, Green RF, Olsson O. Bayes’ theorem and its applications in animal behaviour. Oikos. 2006; 112: 243–251.

[52] Forsman JT, Thomson RL, Seppänen JT. Mechanisms and fitness effects of interspecific information use between migrant and resident birds. Behav Ecol. 2007; 18: 888–894.

[53] Doligez B, Pärt T, Danchin É, Clobert J, Gustafsson L. Availability and use of public information and conspecific density for settlement decisions in the collared flycatcher. J Anim Ecol. 2004; 73: 75–87.

[54] Hamilton WD. Geometry for the selfish herd. J Theor Biol. 1971; 31: 295–311. 5104951 10.1016/0022-5193(71)90189-5

[55] Clark KL, Robertson RJ. Spatial and temporal multi-species nesting aggregations in birds as anti-parasite and anti-predator defences. Behav Ecol Sociobiol. 1979; 5: 359–371.

[56] Sridhar H, Beauchamp G, Shanker K. Why do birds participate in mixed-species foraging flocks? A large-scale synthesis. Anim Behav. 2009; 78: 337–347.

[57] Beehler BM, Foster MS. Hotshots, hotspots and female preference in the organization of lek mating systems. Am Nat. 1988; 131: 203–219.

[58] Hahn BA, Silverman ED. Managing breeding forest songbirds with conspecific song playbacks. Anim Conserv. 2007; 10: 436–441.

[59] Bayard TS, Elphick CS. Testing for conspecific attraction in an obligate saltmarsh bird: can behavior be used to aid marsh restoration? Wetlands. 2012; 32: 521–529.

[60] Battin J. When good animals love bad habitats: ecological traps and the conservation of animal populations. Conserv Biol. 2004; 18: 1482–1491.

[61] Gilroy JJ, Sutherland WJ. Beyond ecological traps: perceptual errors and undervalued resources. Trends Ecol Evol. 2007; 22: 351–356. 17416438 10.1016/j.tree.2007.03.014

