# Supplementary material for: Predation-Related Costs and Benefits of Conspecific Attraction in Songbirds—An Agent-Based Approach
Source: PLoS One. 2015 Mar 19;10(3):e0119132. doi: 10.1371/journal.pone.0119132 (PMC4366215; doi:10.1371/journal.pone.0119132)
Supplement: S3 File — (DOCX) [file pone.0119132.s003.docx]

**File S3. ODD protocol – Model description**

**Predation-related costs and benefits of conspecific attraction in songbirds – an agent-based approach**

**Authors:** Jakub Szymkowiak, Lechosław Kuczyński

**Affiliation:** Department of Avian Biology and Ecology, Institute of Environmental Biology, Faculty of Biology, Adam Mickiewicz University, Poznań, Poland

**Contact:** JSZ: saintdgm@gmail.com or jszym@amu.edu.pl; LK: lechu@amu.edu.pl

*A.1. Overview*

*A.1.1. Purpose*

The overall purpose of this model is to explore predation-related costs and benefits of using a conspecific attraction strategy in the habitat selection process by songbirds. In particular, the goal of this model is to simulate the nest success patterns of Wood Warblers (*Phylloscopus sibilatrix*) that rely on different types of information when choosing breeding sites and breed in a multi-predator landscape of fear; to determine whether local predator community affects profitability of making settlement decisions based on conspecific cues.

*A.1.2. Entities, state variables, and scale*

The simulation space is designed to represent one of the study plots (Wiry Forest) in our study area – the Wielkopolska National Park (western Poland). It is a discrete 2D square grid of 200 $\times$ 200 cells. Each cell represents an empirical distance of 10 m. Hence, the modeled space covers an area of 4 km^2^, which is approximately equal to the area of the Wiry Forest (4.65 km^2^). The simulation space do not contain obstacles or artificial boundaries and is modeled as a torus.

This model includes three types of agents i.e., birds, nests and predators. The number of each type of agent can be varied by the observer. There are three sub-types of bird agents that exhibit different settling strategy, i.e., cue-providers, cue-users and random-settlers. Each bird agent is characterized by the state variable *settling.status* indicating whether it holds a territory (*settling.status* = 1) or is a non-territorial individual (*settling.status* = 0).

The cue-providers represent birds that make settlement decisions based on personal information. During each model initialization, the cue-providers are distributed randomly within a simulation space at least 10 cells away from the nearest neighbor because the mean nearest-neighbor distance between territories of Wood Warblers in the Wiry Forest (Wielkopolska National Park) equals 102.21 m (n = 92 pairs) (Szymkowiak and Kuczyński, Supplementary Material, File S1).

The cue-users represent birds that rely on social information, i.e., birds that make settlement decisions based on the location of other individuals who have already occupied territories. During the model initialization *n* of the cue-users are distributed randomly, however, at least 10 cells away from the nearest cue-provider. After all cue-users are placed in the modeled space, each of them starts prospecting behavior and became territorial after acquiring social cues (see *A.3.3.* for details). Therefore, the cue-users are temporally mobile agents, characterized by two values that change during the prospecting behavior – the location (expressed in Cartesian coordinates) and the movement heading (expressed in degrees). Moreover, the cue-users (as all bird agents) are characterized by the state variable *settling.status* that by default is set to 0, because they are non-territorial individuals at the beginning of the simulation. However, the *settling.status* of a particular cue-user is switched to 1 when an agent establishes territory.

The random-settlers represent birds that settle at random, i.e., birds that do not rely on any specific type of information when making settlement decisions. After both other types of bird agents became territorial, the random-settlers are placed within a simulation space. Although they represent randomly settled individuals, random-settlers are distributed under the following two restrictions: individuals are at least 10 cells away from other random-settlers and at least 20 cells away from the nearest cluster of cue-providers and cue-users. The first restriction results from the average nearest-neighbor distance between Wood Warbler territories in the Wiry Forest (Wielkopolska National Park). The second limitation has to be introduced, because simulations include all bird agent types. Hence, there is a risk that random-settlers in a purely random distribution would be located in the middle of a group of clustered birds (i.e., cue-providers and cue-users), which might influence the results to a great extent and lead to misleading conclusions. It is also important to note that from modeling perspective, both cue-providers and random-settlers are initially distributed similarly within a simulation space. However, in a biological sense, we implicitly assume that cue-providers are birds that rely on personal information, such as their own breeding experience from the previous year, whereas random-settlers make uninformed decisions. Moreover, both agent types could be distinguished because cue-providers are involved in social interactions with cue-users, which is not the case for random-settlers.

The nests are stationary agents and are distributed randomly within the bird agent’s territories. Although each bird agent occupy a territory with a radius of 5 cells (representing an empirical distance of 50 m), nests are sprouted randomly within a radius of 3 cells measured from the territory center. This approach is intended to account for the mean empirical distance between the territory center and nest location, which equals 32.45 m (n = 47) in our study plot (Szymkowiak and Kuczyński, Supplementary Material, File S1).

Two sub-types of predator agents were simulated in this model, Wild Boar (*Sus scrofa*) and Red Fox (*Vulpes vulpes*), which differed in their foraging behavior. During the model initialization *n* of the Wild Boars and *n* of the Red Foxes are distributed randomly. Both, the Wild Boars and Red Foxes are mobile agents; therefore are characterized by two variables that can change during the simulation i.e., the location (expressed in Cartesian coordinates) and the movement heading (expressed in degrees). Moreover, the Wild Boars are characterized by two fixed values that may be varied by the observer and two state variables. The *boars.activity* and *sleep.time.boars* state variables determine the number of time steps in which boars are involved in foraging and resting behavior, respectively. The *boars.activity.duration* parameter determines the time which the Wild Boars spend on foraging during each day, while the *sleep.duration.boars* parameter refers to the time of boars resting behavior. Because the time of the entire simulation was scaled based on data for the average distance moved by Red Foxes during foraging bouts (see *A.1.3*) and both predators differ in patterns of daily activity, this time differentiation ensures realistic simulation of the Wild Boars foraging behavior (see *A.3.3.4.* for details). The Red Foxes are characterized by two state variables i.e., the *predated.nests* and *ARS.countdown*, and one fixed variable the *duration.ARS.fox*. Those variables determine how long a fox agent spends on performing area-restricted searches (described in *A.3.3.5.*) after depredating a nest. The p*redated.nests* and *ARS.countdown* variables are initially set to 0, while the *duration.ARS.fox* variable may be varied by the observed before the model initialization or during the simulation. When a fox agent depredates a nest, the value of the *predated.nests* variable increases by a unit and the value of the *ARS.countdown* variable increases by the value of the *duration.ARS.fox*. Then, a fox starts to perform area-restricted search and in each time step the value of the *ARS.countdown* decreases by a unit. When the *ARS.countdown* equals 0, a fox switch foraging strategy to a standard correlated random walk (see *A.3.3* for details about both movement behaviors) and the value of the *predated.nests* variable is set to 0 again.

*A.1.3. Process overview and scheduling*

Each run of this model consists of two stages: (1) the simulation of birds settling and (2) predators foraging behavior. In the first stage, after the model initialization the cue-providers and cue-users are randomly distributed within the simulation space. Then, the cue-users start prospecting behavior and stop (settle) after acquiring an information about location of birds that have already occupied territories. During prospecting, the cue-users are scheduled for move in ascending order based on the ID number. When each cue-user occupies territory, the random-settlers are placed in the simulation space.

Note: For this model stage, time is not specified because we did not aim to investigate temporal dynamics of conspecific attraction mechanism *per se*; but rather model two variants of this behavior and study fitness consequences of clustered distribution that emerges as a by-product of relying on conspecific cues. In nature, however, the time-frame for territory selection in songbirds may potentially be short, thereby reducing time which birds can spend on updating of prior settlement decisions.

After all birds are settled the simulation of predators foraging behavior is performed, in which time moves forward in a discrete steps of fixed size i.e., 1 tick per step. Considering the following: (1) predators move forward 1 grid cell per time step; (2) in nature, foxes often move ~2 km per foraging bout (Phillips et al., 2004, Ringelman 2014); and (3) a single cell represents an empirical distance of 10 m; we defined one day as 200 time steps. Because the nest cycle of Wood Warblers lasts 32 days, all simulations are run for 6400 time steps. Predator agents are scheduled for performing foraging behavior in ascending order based on the ID number. During each step, the predators move and eat the nests if they are occupying the same cell. The nest agents perform no action or do change color if they are detected by a predator, and by this they become unavailable for other predators.

*A.2. Design concepts*

*A.2.1. Basic principles*

Birds use various types of information about habitat when deciding where to settle. Some of them follow a trial-and-error strategy and choose between concurrent areas based on personal information, while other rely on social information i.e., observe and copy the decisions made by con- or heterospecifics (Danchin et al., 2004; Dall et al., 2005). Those different strategies of making settlement decisions lead to various patterns of spatio-temporal distribution of individuals. Songbirds that follow a conspecific attraction strategy in the habitat selection process prefer to settle in habitat patches already occupied by other individuals which leads to clustered breeding. Because predators may respond functionally to prey distribution, this likely affects the potential costs and benefits of using a particular habitat selection strategy in a multi-predator landscape. Moreover, when relying on social information, birds are involved in intra- and interspecific interaction between individuals which make decisions based on social cues (the cue-users) and those that are cues for others (the cue-providers). Perhaps also the outcome of this interaction may be shaped by some predation-related costs and benefits. However, this constitutes a large gap in our knowledge due to issues with collecting appropriate empirical data (Szymkowiak, 2013). This model is designed to allow for a better understanding of the economy of using social information in the habitat selection process by songbirds.

*A.2.2. Emergence*

Two emergent patterns appear in this model. Firstly, clustered distribution of the cue-provider and cue-user territories. This is a consequence of simply rules that the cue-users follow when making settlement decisions. During prospecting behavior the cue-users move independently from each other and decide where to settle after acquiring specific type of social cue i.e., the information about location of other individuals that have already occupied the territory. Hence, clustered distribution of the cue-provider and cue-user territories arises from the interaction between those two bird agent sub-types. The second emergent property that appears in this model is the different nest success between nests that are differently distributed. In this model, the nest success is calculated as the percentage of non-depredated nests of each bird agent sub-type at the end of the simulation. The emergent pattern appears from the interaction between the predator behavior and different types of nest distribution (either clustered or randomly spaced).

*A.2.3. Adaptation*

The cue-users make settlement decisions based on conspecific cues i.e., follow the rule according to which they choose breeding sites based on location of other individuals that have already occupied territories (*A.3.3.1.* and *A.3.3.2.*). In some simulations, the cue-providers and cue-users perform Bayesian updating of prior settlement decisions and avoid settling in areas with too many neighbors. The Red Foxes normally move in a correlated random walk, but after encountering a nest they switch the movement strategy and perform more detailed searching of nearby area (*A.3.3.5.*). The stationary nests and random-settlers, as well as Wild Boars that move only in a correlated random walk when foraging do not implement any adaptive behavior.

*A.2.4. Objectives*

The cue-users change their behavior after acquiring a social cue in a way that could increase their chances for finding a high-quality habitat by “assuming” that occurrence of other individuals in a particular area is positively correlated with its quality. The cue-providers and cue-users that perform Bayesian updating try to avoid the risk of clustering with too many neighbors. The Red Foxes change movement strategy after encountering a nest and perform area-restricted search that may increase their chances for finding another food resource near the previous one if the nest distribution is spatially autocorrelated.

*A.2.5. Learning*

Agents do not have any ability for learning in this model.

*A.2.6. Prediction*

The cue-providers and cue-users that perform a Bayesian updating strategy (see *A.3.3.2.*) implicitly predict that nesting in large clusters may increase predation risk, hence try to avoid settling near too many neighbors. The Red Foxes change foraging behavior after encountering a nest (see *A.3.3.5.*) which is based on implicit assumption about higher probability of encountering other nest nearby already located food resource.

*A.2.7. Sensing*

In the basic conspecific attraction process (see *A.3.3.1.*), the cue-users are able to acquire information about location of other bird agent that occupies territory in a radius of 10 cells, which equates to a detection range of $10\times\sqrt{100+10}0\approx$ 141.4 m. Biologically, we assumed here that a particular bird has ability to hear another individual from this distance which should not constitute a cognitive issue for the Wood Warblers, because a singing male can be heard from such distance even for the human-observer (pers. obs.). In another set of simulations, birds performed more sophisticated mechanism of conspecific attraction (see *A.3.3.2.*). The cue-providers and those cue-users that acquired social cue assessed the number of other individuals in a radius of 15 cells prior to making final settlement decision, which equates to a detection range of $15\times\sqrt{100+10}0\approx$ 212.1 m. The predators are able to detect the nest if they are occupying the same cell which equates to a detection range of $\sqrt{100+10}0\approx$ 14.1 m, which is a reasonable range for the mammalian predators (Österholm, 1964; Seymour et al., 2004).

*A.2.8. Interaction*

The cue-users make settlement decisions based on location of birds that have already occupied territories, hence interact with them directly. The cue-providers and cue-users when performing Bayesian updating interact with other territorial birds in a 15-cell buffer. Each predator agent interacts directly with nests by foraging on them, and also indirectly (indirect competition) with other predators which exploit the same food resources.

*A.2.9. Stochasticity*

Stochasticity appears in this model at two levels. Firstly, all agents are distributed randomly within the simulation space after model initialization. Secondly, mobile agents move in a correlated random walk with a randomly chosen heading drawn from a normal distribution with a mean of 0° and a standard deviation that may be varied by the observer.

*A.2.10. Collectives*

The cue-users settle near birds that have already occupied territories. This leads to clustered distribution of the cue-user and cue-provider territories and the nests, which may be considered as collectives.

*A.2.11. Observation*

At the end of each simulation run (6400 time steps), the number of nests that remained non-depredated was noted. Based on this data, we calculated the nest success for each bird agent sub-type as the percentage of non-depredated nests in relation to the initial number of nests. This metric was used in further analysis.

*A.3. Details*

*A.3.1. Initialization*
 The model was initialized with 80 total bird agents (and their nests), which is equivalent to the density of breeding Wood Warbler pairs in the Wiry Forest of the Wielkopolska National Park. Wood Warblers exhibit little site tenacity, with a median of 11% of males returning to previous breeding areas (n = 4 publications, based on Table 1 in Wesołowski et al., 2009). We assumed that these males rely on personal information when making settlement decisions; thus, 8 cue-providers were initially placed in the simulation space. Among the remaining 72 bird agents, 36 were modeled as cue-users and 36 as random-settlers. Different settling strategies of cue-users were modeled in different simulations. Hence, in a particular simulation run, cue-users followed either basic conspecific attraction strategy (see *A.3.3.1.*) or a conspecific attraction with Bayesian updating (see *A.3.3.2.*). A total number of 6 predators were modeled, either in the pure as well as in the mixed predator community.

*A.3.2. Input data*

This model does not use any input data.

*A.3.3.* *Submodels*

Each run of this model consists of two stages – the simulation of birds settling and predators foraging behavior. The cue-providers and random-settlers are distributed at random within the simulation space. The cue-users follow one of two conspecific attraction strategies when making settlement decisions, described as submodels *A.3.3.1.* and *A.3.3.2.* The bird agents build nests after all birds are settled. The predators foraging behavior consists of three submodels: the Wild Boar foraging, the Red Fox foraging and the nest eating.

*A.3.3.1.* The c*onspecific attraction submodel*

In this submodel, the cue-users follow a basic conspecific attraction strategy when making settlement decisions. Therefore, the cue-users “assume” that occurrence of other individuals in a particular place is positively correlated with its quality and decide where to settle based on location of conspecifics. When all cue-users are placed in the simulation space, each of them starts to move in a correlated random walk (one cell per time step), which represents prospecting behavior. The direction of a movement is drawn at each time step, from a normal distribution with a mean 0° and a standard deviation defined by the observer using the *turning.cue-users* parameter. In our simulations, we set *turning.cue-users* = 15, as preliminary model exploration revealed that this setup facilitate the time of finding conspecifics. However, this parameter may be set freely by the observer, as it does not affect the results of predator foraging simulations, but only influences the duration of the conspecific attraction submodel execution. During prospecting, at each time step, non-territorial cue-users are asked whether there is another bird agent in a radius of 10 cells, that have already occupied territory (i.e., has *settling.status* = 1). If another agent is present, the cue-user perceives this information as a social cue, stops, switches own *settling.status* from 0 to 1 and becomes territorial. If there is no such individual, the cue-user continues prospecting behavior. This submodel ends when no single cue-user has *settling.status* = 0 i.e., when all cue-users occupy territories.

*A.3.3.2. The conspecific attraction with Bayesian updating submodel*

In this submodel birds perform more sophisticated conspecific attraction strategy, that includes a form of Bayesian updating of prior settlement decisions. Firstly, each cue-user follows basic conspecific attraction strategy as described in *A.3.3.1.* However, at each time step, all cue-providers and those cue-users that acquired social cues (and are settled) are asked how many other individuals are in a radius of 15 cells. If the number exceeds a threshold value defined by the observer (*k* parameter), bird agents move (one cell per time step) according to a correlated random walk with a heading drawn from a normal distribution (mean = 0, SD = 360). Because the turning angle is high, this results in a very tight, convoluted movement that is continued until the number of individuals in a radius of 15 cells is equal to or lower than the threshold value. If this assumption is fulfilled, the bird agent stops immediately. Because the birds are asked to assess the number of other territorial individuals in a 15-cell buffer at each time step, this “updating of territory borders” could be induced repeatedly if there are newly arriving birds at a particular location. This procedure is completed after all cue-providers and cue-users occupy stable territories. In a biological sense, we model behavior in which the cue-users follow a conspecific attraction strategy; however, together with the cue-providers, they also perceive nesting in clusters with many other birds as a potential threat. This may be because large clusters could facilitate the localization of prey by predators (Krause and Godin, 1995; Ioannou and Krause, 2008); thus, individuals try to avoid settling near too many neighbors. Therefore, this behavior is a form of Bayesian updating in which birds adjust prior settlement decisions based on information about the social environment obtained *a posteriori*.

It is important to note that the actual model code do not completely preclude the scenario in which two birds randomly move simultaneously without increasing their distance from each other for a certain time period; however, this behavior has the potential to spread the cluster farther, which might amplify these results. However, the probability of such scenario seems to be minor, as turning angle of the movement during Bayesian updating is high (SD = 360) which results in a movement path being very tortuous and an individual does explore the area near its current location. Therefore, it is likely that such “connected” individuals would quickly be separated as they would find an optimal location soon and before moving too far from the cluster; hence simultaneous movement probably would quickly vanish.

*A.3.3.3.* *The nest building submodel*

When all birds in the simulation space are territorial, one nest is sprouted within a territory of each bird agent i.e., within a radius of 3 cells measured from the territory center.

*A.3.3.4.* *The* *Wild Boar* *foraging submodel*

The Wild Boars are incidental predators of songbird nests and move in a correlated random walk during foraging bouts. The direction of a movement is drawn at each time step from a normal distribution with a mean 0° and a standard deviation defined by the observer using the *turning.boars* parameter. At each time step predator moves forward one grid cell. In nature, the Wild Boars and Red Foxes move on a different distance during foraging bouts, which is twice less for the Wild Boars than Red Foxes. The time of the entire simulation is scaled based on data for the average distance moved by Red Foxes during foraging bouts. Hence, there are two parameters that limit the Wild Boars activity to account for different patterns of daily activity of both predators. At each time step, the state variable *boars.activity* increases by one unit if Wild Boars perform foraging. When the *boars.activity* variable equals the number of the *boars.activity.duration*, an observer-defined parameter, boars stop moving and perform resting behavior. The duration of the Wild Boars’ resting behavior is defined by the observer using the *sleep.duration.boars* parameter. When boars rest, the state variable *sleep.time.boars* increases by one unit at each time step. If the *sleep.time.boars* variable equals the *sleep.duration.boars* parameter, the value of both Wild Boars’ state variables are set to 0 and predators start foraging behavior again. In our model, one day is defined as 200 time steps, which allows for Red Foxes to move on 2 km distance per day (see *A.1.3.*). At the same time, the Wild Boars are expected to move on distance ≈ 1 km (Diong, 1982), therefore both, the *boars.activity.duration* and *sleep.duration.boars* parameters are defined by default as 100 time steps.

*A.3.3.5. The Red Fox foraging submodel*

The Red Fox represented a predator with a capacity to perform more intensive searching of nearby areas after encountering a nest. Normally, foxes moved in a correlated random walk with a movement heading that was drawn, at each time step, from a normal distribution with a mean of 0 and a standard deviation defined by the observer using the *turning.foxes* parameter. However, foxes changed their movement strategy and performed area-restricted searches (ARSs) after encountering a nest. During the ARS, direction of a movement is drawn from a normal distribution with a mean of 0° and the observer-defined standard deviation given by the *strength.ARS.fox* parameter. Because the value of the *strength.ARS.fox* parameter is higher than the value of the *turning.foxes* parameter, predator’s turning angle increases in the ARS movement and foxes explore nearby area instead of moving further away. The duration of the ARS movements is controlled by the *duration.ARS.fox* parameter, defined by the observer. If the fox agent does not find any other nests during this time, it resumes movement in a standard correlated random walk.

*A.3.3.6. The nest eating submodel*

The Wild Boars and Red Foxes are able to eat nests if they are occupying the same cell. After being eaten, nest changes color and becomes no longer available for the predators to the end of the simulation.

**Appendix A, Literature Cited**

Dall, S.R.X., Giraldeau, L.A., Olsson, O., McNamara, J.M., Stephens, D.W., 2005. Information and its use by animals in evolutionary ecology. Trends Ecol. Evol. 20, 187-193.

Danchin, E., Giraldeau, L.A., Valone, T.J., Wagner, R.H., 2004. Public Information: from nosy neighbors to cultural evolution. Science 305, 487-491.

Diong, Ch.D., 1982. Population biology and management of the feral pig (Sus scrofa, L.) in Kipahulu Valley, Maui. PhD thesis.

Glutz von Blotzheim, U.N., Bauer, K.M., 1991. Handbuch der Vögel Mitteleuropas. AULA-Verlag, Wiesbaden.

Ioannou, C.C., Krause, J., 2008. Searching for prey: the effects of group size and number. Anim. Behav.75, 1383-1388.

Krause, J., Godin, J.G.J., 1995. Predator preferences for attacking particular prey group sizes-consequences for predator hunting success and prey predation risk. Anim. Behav. 50, 465-473.

Österholm, H., 1964. The signiﬁcance of distance receptors in the feeding behavior of the fox *Vulpes vulpes* L. Acta Zool. Fennica 106, 1–31.

Phillips, M.L., Clark, W.R., Nusser, S.M., Sovada, M.A., Greenwood, R.J., 2004. Analysis of Predator Movement in Prairie Landscapes with Contrasting Grassland Composition. J. Mammal. 85, 187–195.

Ringelman, K.M., 2014. Predator foraging behavior and patterns of avian nest success: What can we learn from an agent-based model? Ecol. Model. 272, 141–149.

Seymour, A.S., Harris, S., White, P.C.L., 2004. Potential effects of reserve size on incidental nest predation by red foxes *Vulpes vulpes*. Ecol. Model. 175, 101–114.

Szymkowiak, J., 2013. Facing uncertainty: how small songbirds acquire and use social information in habitat selection process? Springer Science Reviews 1, 115-131.

Wesołowski, T., Rowiński, P., Maziarz, M., 2009. Wood Warbler *Phylloscopus sibilatrix*: a nomadic insectivore in search of safe breeding ground? Bird Study 56, 26-33.
